# The Relationship Between the Contact Force at the Ankle Hook and the Hamstring Muscle Force During the Nordic Hamstring Exercise

**DOI:** 10.3389/fphys.2021.623126

**Published:** 2021-03-09

**Authors:** Mianfang Ruan, Li Li, Weiping Zhu, Tianchen Huang, Xie Wu

**Affiliations:** ^1^Sports Biomechanics Laboratory, College of Physical Education and Health, Wenzhou University, Wenzhou, China; ^2^Department of Health Sciences and Kinesiology, Georgia Southern University, Statesboro, GA, United States; ^3^School of Kinesiology, Shanghai University of Sport, Shanghai, China

**Keywords:** Nordic hamstring exercise, eccentric strength, Nordbord, 3D kinematics, OpenSim

## Abstract

A novel device has been developed to assess eccentric hamstring strength during the Nordic hamstring exercise (NHE) by measuring the contact force at the ankle hook (brace). The purpose of this study was to determine the correlation between the force measured at the ankle hook and the hamstring force estimated by a low extremity model. Thirteen male college sprinters were recruited to perform NHE on an instrumented device Nordbord (Vald Performance, Australia). Contact forces were measured at a sampling rate of 50 Hz at the hooks using the uniaxial load cells. 3D kinematics were measured simultaneously at a sampling rate of 200 Hz using a 16-camera motion analysis system (Vicon Motion Analysis, Oxford, United Kingdom) during the NHE. The data were processed with Visual 3D (C-Motion, Germantown, MD, United States) and OpenSim (NCSRR, Stanford, CA, United States) to calculate the knee joint center’s coordinates and hamstring moment arms during NHE. A static low extremity model was built to estimate the hamstring force during NHE. We have observed a significant but not very high correlation (*r*^2^ = 0.58) between peak hamstring force and the peak contact force at the ankle hook. The peak contact force measured at the ankle hook can only explain a little more than half of the variations in peak hamstring muscle forces during NHE. Caution must be exercised when assessing the eccentric hamstring strength using the ankle contact force during NHE.

## Introduction

Hamstring strain injuries (HSIs) are prevalent in different sports (Crema et al., 2018), especially in sports involving sprinting (Askling et al., 2007), such as track and field (Crema et al., 2018), Australian football (Orchard et al., 2013), and soccer (Ekstrand et al., 2016). Several studies regarding the mechanism and risk factors associated with HSIs have been published (Li and Wang, 2017; Liu et al., 2017; Yu et al., 2017). It is generally accepted that these types of injuries are multi-factorial. Although HSIs’ direct cause remains unknown (Ruan, 2018), either overstrain or overstress or both during highspeed eccentric contractions could be the specific mechanical parameter that causes injury. The use of eccentric strength exercise in preventing HSIs has been advocated by many studies (Guex and Millet, 2013; Opar et al., 2012). The Nordic hamstring exercise (NHE), an eccentric exercise easy to implement, is widely promoted since it effectively reduces HSIs (van der Horst et al., 2015).

Hamstring weakness is one of the most common risk factors associated with hamstring injuries (Jonhagen et al., 1994; Askling et al., 2003; Sugiura et al., 2008). Hamstrings need to produce sufficient force to decelerate the sw**i**ng leg or counteract external contact torques (caused by the ground reaction force at the initial contact) during sprinting (Sun et al., 2015). It was proposed that the hamstring strength exercises used should be specific to simulate muscle-tendon length and the high load eccentric contractions at the knee joint developed by the hamstring (Guex and Millet, 2013). Although NHE’s effect on HSIs prevention is well documented, the effect of the NHE on hamstring eccentric strength, as seen during sprinting, remains unclear (Milanese and Eston, 2019). The main reason for this is none of the existing studies have estimated the hamstring force that occurs during the NHE, making it difficult to determine whether the hamstring force that occurs during the NHE could simulate the high muscle force observed during sprinting (Li and Ruan, 2018; Ruddy et al., 2018a). A Novel device has been developed to assess the eccentric hamstring force during the NHE (Opar et al., 2013) and a few studies (Buchheit et al., 2016; Ruddy et al., 2018b) using data from this device have been published. However, it remains a question whether the contact force measured by load cell at the ankle hook (brace) could reflect the hamstring force that occurred during the NHE.

This study’s purposes were to develop a muscle model to estimate the hamstring force during NHE; and to determine the correlation between the force measured at the ankle hook and the hamstring force estimated by the muscle model.

## Methods

### Participants

Thirteen male college sprinters (mean ± SD, age: 20.3 ± 1.1 years; height: 1.81 ± 0.72 m; body mass: 71.5 ± 7.7 kg; personal bests in 100 m rush: 11.2 ± 0.3 s) who had received no less than 5 years of sports training participated in the study. The participants were eligible for inclusion if they did not have a history of lower limb injury within the previous 12 months and had never sustained a hamstring strain injury. All participants were experienced in the Nordic exercise. The Ethics Committee of Shanghai University of Sport approved the project, and participants signed informed consent forms before participation. Written informed consent was obtained from the individual for the publication of any potentially identifiable images or data included in this article.

### Procedure

**FIGURE 1:**
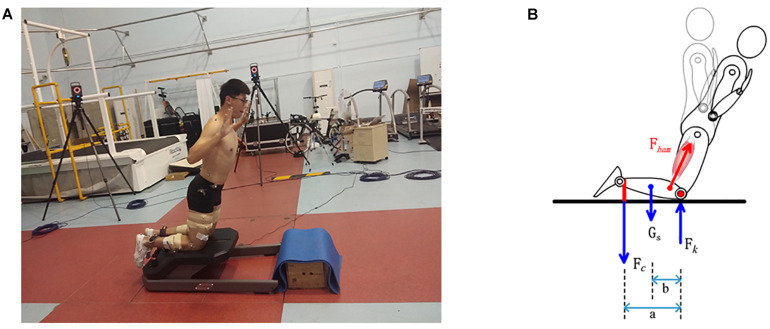
**(A)** Marker set up and the instrumented device (Nordbord) for assessing Nordic hamstring exercise. **(B)** Free body diagram for the shank during Nordic hamstring exercise.

The participants performed the NHE on an instrumented Nordbord (Vald Performance, Australia). The details of the instrument have been reported previously (Opar et al., 2013). Retroreflective markers (14 mm) were attached bilaterally on participants’ acromioclavicular joints, ilium crest tubercle, anterior superior iliac spines, posterior superior iliac spines, greater trochanters, medial and lateral epicondyles of the knee joint, medial and lateral malleoli, the first and fifth metatarsal heads, posterior surface of calcaneus, and the second toes. Additional rigid plates with 3 markers were attached bilaterally to the thighs and shanks (Figure 1A). Participants performed a static calibration trial with all markers presented. The calibration markers, including the greater trochanters, medial and lateral epicondyles of the knee joint, and medial and lateral malleoli, were then removed before the warm-up. For the assessment of the eccentric hamstring force, participants were instructed to kneel on the padded part of the NordBord, with the ankle joints secured with padded hooks, which were affixed atop uniaxial load cells (Figure 1A). The hooks and load cells were mounted on a pivot, which allowed the load cells to be perpendicular to the shank at all times. Retroreflective markers were also attached to the hooks.

The participants began with a standard warm-up of running on a treadmill for 5 min. Then, participants performed two sets of 5 NHE movements with a 2 min interval between each set. As the starting position, participants kneeled on the padded part of the NordBord with the upper body vertical and straight, and the ankle joints were secured with padded hooks. Participants were required to gradually lean forward by contracting the hamstrings and keeping the trunk and hips held in a neutral position throughout (Buchheit et al., 2016). Participants’ arms were flexed at the elbow joints and the palms of the hands were facing forward at the level of the shoulder joints. In the final stages of the movement, participants were allowed to use their arms to buffer the downward movement. Then, participants pushed the ground and returned the body to the initial kneeling position. A metronome was used to control the downward duration lasting about 3 s. A rejected repetition included failing to maintain trunk and hip in the neutral position or failing to control the descent at the beginning of the movement. Contact forces were measured at a sampling rate of 50 Hz at the hooks using the uniaxial load cells. 3D kinematics were measured simultaneously at a sampling rate of 200 Hz using a 16-camera motion analysis system (Vicon Motion Analysis, Oxford, United Kingdom) during the NHE. The repetition with the highest contact force in the second set was chosen for later analysis.

### Data Reduction

The raw data were processed with a 3D biomechanical analysis suite, Visual 3D (C-Motion, Germantown, MD, United States), to compute the 3D kinematic variables. The 3D marker coordinates were smoothed using a fourth-order Butterworth low-pass filter with cutoff frequencies of 10 Hz (Winter, 1990). They were then processed with OpenSim (NCSRR, Stanford, CA, United States) to calculate the knee joint center’s coordinates and hamstring moment arms during NHE.

### Hamstring Force Estimation

As showed in Figure 1B, the free body diagram for shank was used to estimate the hamstring force. The forces that must be included on the free body diagram are the gavity force of the system, the musculoskeletal force and the load due to the apparatus (Enoka, 2008). The knee joint was modeled as a one-degree of freedom joint in which only sagittal rotations were allowed. We assume that the F_k_ was cross the knee joint center and did not generated any moment, and only the hamstring muscles generated the knee joint flexion moment. Since participants gradually leaned forward during NHE knee angular acceleration is close to zero, it was referred to as a static condition (Enoka, 2008). In a static analysis, the sum of the moments in the clockwise direction is equal to the moments in anti-clockwise direction. Therefore, we can derive the following equations:

(1) $${\text{M}}_{o\,\left(F_{\text{ham}}\right)}=M_{o\,\left(F_{c}\right)}+M_{o\,\left(G_{s}\right)}$$

(2) $$F_{\text{ham}}\times l=F_{c}\times a+G_{s}\times b$$

Where M_o(Fham)_ is the moment generated by hamstring about knee joint center O; M_0(__*Fc)*_ is the moment generated by contact force F_c_ about knee joint center O; *M*_o(Gs__)_ is the moment generated by gravity force of the shank (G_s_) about knee joint center torque*; l* is the instant hamstring moment arms; *a* is the moment arm of Fc; b is the moment arm of Gs; F_c_ is the contact force at ankle hook measured by Nordbord, and *a* was calculated by subtracting the horizontal coordinates of the marker on the ankle hook to the horizontal coordinates of the knee joint center. The relative weight of the shank and foot, to body weight (BW), was estimated based on Winter (Winter, 1990): Gs = 0.061 × BW. The b was calculated from Visual 3D model: *b* = 0.606 × Length of the shank.

**FIGURE 2:**
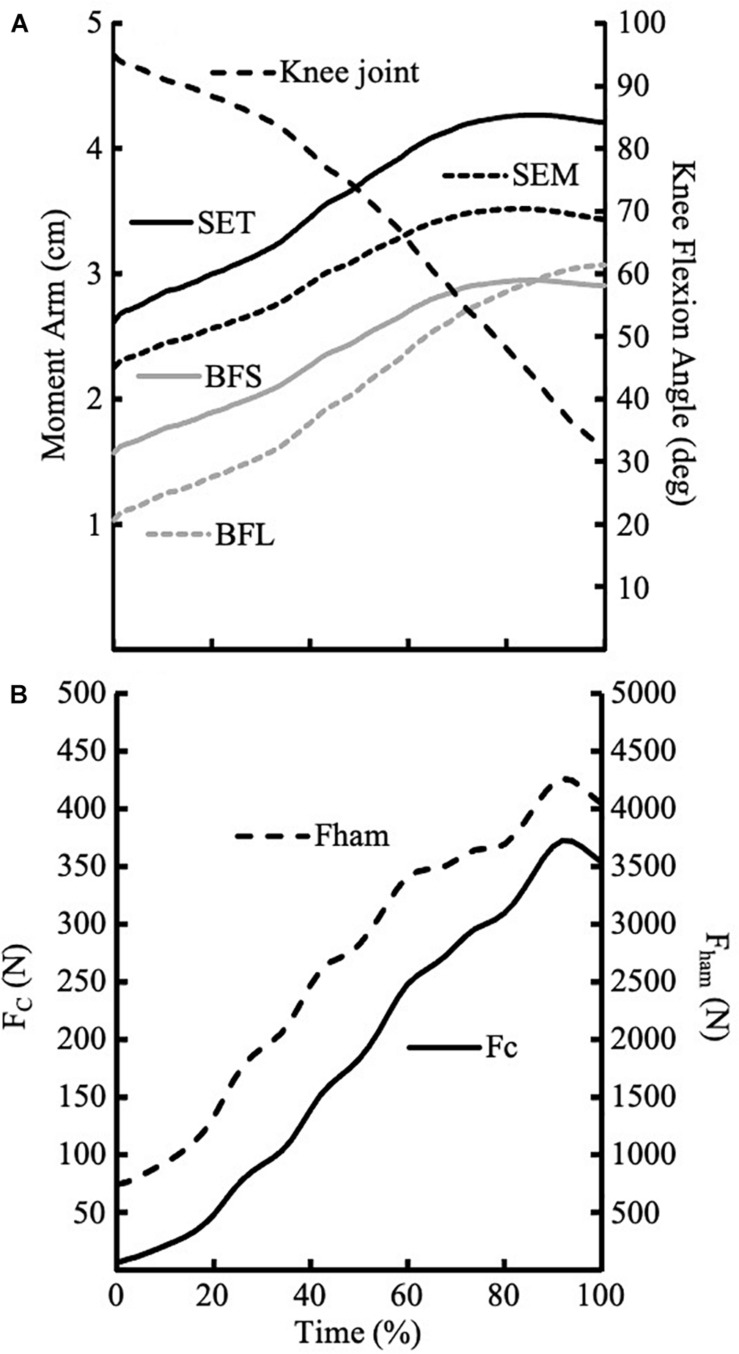
**(A)** Exemplar traces of moment arms for four muscles during Nordic hamstring exercise from one participant. The knee joint angle was flexed at 90° at the beginning of the trial and the decreasing to about 35° knee flexion at the end of the trial. **(B)** Exemplar traces of F_c_ and F_ham_ during Nordic hamstring exercise from one participant.

Following the 3D marker coordinates were processed by Visual 3D, mot files were input to OpenSim (NCSRR, Stanford, CA, United States) to estimate the hamstring moment arms. Figure 2A shows exemplar changes of moment arms of the four hamstring muscles during Nordic hamstring exercise.

As shown in Figure 2A, the moment arms are different for the four hamstring muscles. We assumed the ratio of force distribution among four muscles was correlated to their maximum isometric force which was determined by the physiological cross-sectional area of the muscle. The force distribution data were provided by OpenSim: BFL(Biceps Femoris long head) = 0.26; BFS (Biceps Femoris short head) = 0.24; SEM(semimembranosus) = 0.12; SET(semitendinosus) = 0.38.

Using the force distribution data, we can drive the following equation:

$$F_{\text{ham}}\times l=0.26\times F_{\text{ham}}\times B\,F\,L_{a\,r\,m}+0.24\times F_{\text{ham}}\times B\,F\,S_{a\,r\,m}+0.12\times F_{\text{ham}}\times S\,E\,M_{a\,r\,m}+0.38\times F_{\text{ham}}\times SET_{a\,r\,m}=F_{\text{ham}}(0.26BFL_{a\,r\,m}+0.24BFS_{a\,r\,m}$$

(3) $$+0.12SEM_{a\,r\,m}+0.38SET_{a\,r\,m})$$

Where *BFL*_*arm*_ is the moment arm of BFL; *BFS*_*arm*_is the moment arm of BFS; *SEM*_*arm*_is the moment arm of SEM; *SET*_*arm*_ is the moment arm of SET. The hamstring forces can be estimated using Eqs 2 and 3.

### Statistics Analysis

Descriptive statistics of Fc and Fham were presented as mean, standard deviation, minimum and maximum values. A Pearson’s correlation coefficient r was used to determine relationships between peak values of F_c_ and F_ham_. Where 0.36 < *r* < 0.67 (0.13 < *r*^2^ < 0.45), as moderate correlation, 0.68 < *r* < 0.9 (0.46 < *r*^2^ < 0.81), as high correlation, *r* = 0.9 (*r*^2^ > 0.81), as very high correlation (Taylor, 1990). An alpha level of p < 0.05 was considered statistically significant. Statistical analysis was performed using SPSS software (version 19.0; SPSS, Chicago, IL, United States).

## Results

Figure 2A shows exemplar traces of the moment arms of four muscles during the NHE where Figure 2B shows the exemplar data of F_c_ and F_ham_. Table 1 shows the peak values of F_c_ (pF_c_) and F_ham_ (pF_ham_), and knee joint angles at the peak values.

**TABLE 1:** Force and angle results of the Nordic hamstring exercise.

	Mean	*SD*	Min	Max
pF_c_ (N)	314.0	67.9	241.7	421.4
pF_c_/body weight	0.45	0.10	0.33	0.63
pF_ham_ (N)	3734.8	678.9	2587.4	4993.5
pF_ham_/body weight	5.43	1.13	3.92	7.49
Angle at pF_ham_(deg)	48.8	17.6	19.0	71.4
Angle at pF_c_(deg)	46.8	15.0	23.9	70.4

**FIGURE 3:**
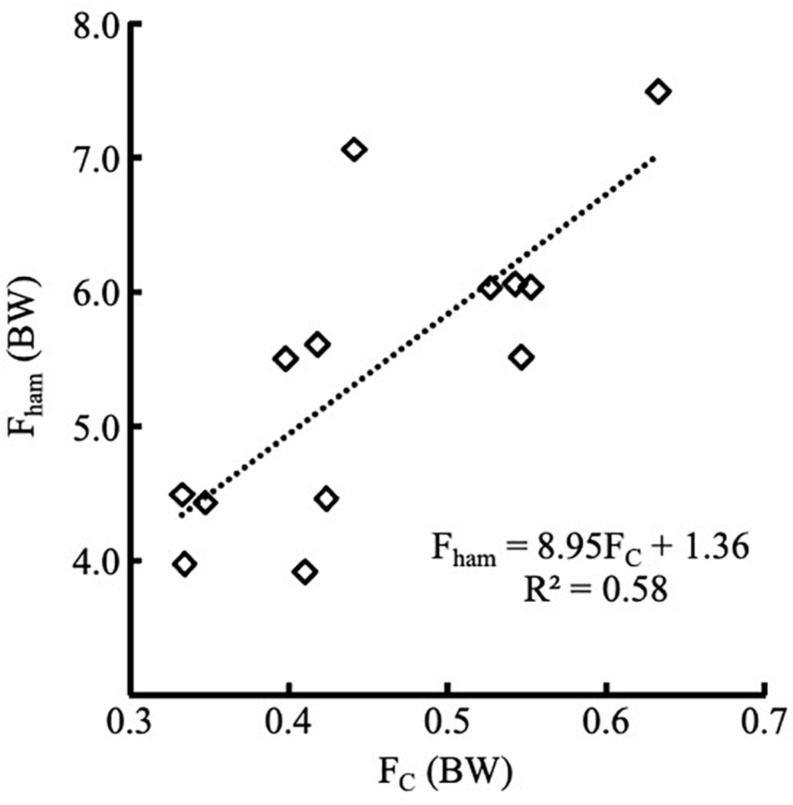
Linear positive relationships between body weight normalized F_c_ and F_ham_ (*p* < 0.01). F_ham_ = 8.95*F_c_+1.36, *r*^2^ = 0.58.

There was a significant correlation (*r*^2^ = 0.58) between pF_c_ and pF_ham_ (see Figure 3 for more details).

## Discussion

To our knowledge, this is the first study to estimate the hamstring force produced during the NHE and compare it with the ankle contact force measured by the Nordbord. The ankle contact force has been used to directly evaluate the eccentric hamstring force (Opar et al., 2013). It was a convenient field-based method to assess eccentric hamstring strength. However, we found that the ankle contact force at hook is not equal or linear to the hamstring force. Actually, the moment generated by the ankle contact force is approximately equal to the moment generated by the hamstring force (the moment generated by gravity of the shank could be ignored because it is very small.) The authors (Opar et al., 2013) might have confused moment with force. We observed a significant, but not very high correlation (*r*^2^ = 0.58) between peak hamstring force and the peak contact force at ankle hook.

A linear regression equation was established to predict the pF_ham_ using pF_c_. However, only about 58% (coefficient of determination) of peak hamstring forces could be explained by the peak contact force measured at the ankle hook. Other factors also contribute to the prediction of pF_ham_. First, the knee joint angles at which the maximum hamstring force occurred varied between 71.37 and 18.97 deg. Accordingly, a large inter-individual difference existed in the instant moment arms of peak hamstring force among participants. Although we have used a metronome to control the downward movement’s pace, the kinematic data still show considerable variability. Second, *a* (the moment arm of F_c_) was different (about 5%) among participants, which could be attribute to the difference in the length of shank among participants.

Ruddy and colleagues (Ruddy et al., 2018b) build a supervised learning (a type of machine learning) model, which included eccentric hamstring strength (peak value of F_c_ during NHE), age, and previous HSI, to predict the occurrence of HSI. Unfortunately, the predictive performance was just slightly better than random. We argued that NHE’s eccentric strength does not reflect the high eccentric force during sprinting (Li and Ruan, 2018). Another report (Sun et al., 2015) calculated the different joint torque components via an intersegmental dynamics approach. It estimated that the peak hamstring force that occurred during sprinting (9.7 ± 0.3 m/s) ranged from 5,777 to 11,554 N, or at least 8 times of body weight. Schache et al. (2012) calculated hamstring force strain during sprinting (8.9 ± 0.7 m/s) via an optimization algorithm and reported that the peak hamstring force was about 9 times of body weight. The current study showed that the peak hamstring force during NHE was 5.43 ± 1.13 times of body weight. The results supported our previous argument. Overall, peak hamstring force occurred during NHE was not comparable to the peak hamstring force occurred during sprinting. Furthermore, the contact force measured at the ankle hook (F_c_) can only explain 58% of hamstring force. Therefore, it is not surprised that the predictive performance using F_c_ was just slightly better than random.

There are a few limitations to our study. Firstly, the data of muscle moment arms are provided by OpenSim rather than *in vivo* measurement. Therefore, some deviation may exit. Fortunately, the magnitudes of muscle moment arms provided by OpenSim are within the range of values from an *in vivo* measurement (Kellis and Baltzopoulos, 1999). Secondly, we assumed the force distribution among the four hamstring muscles was correlated to their maximum isometric force and did not consider the muscle activation level among different muscles. While Delahunt et al. (2016) reported that there was no significant difference in activity between BF and ST when compared to MVC, van den Tillaar et al. (2017) observed that the activation levels in ST reached almost 70% compared with BF of only 40% when compared to the peak values during sprinting. If we assume the force distribution among four muscles was correlated to their maximum isometric force and muscle activation level, and the moment arms of BFL, BFS, SEM, and SET are 1.5, 2.0, 2.8, and 3.5 cm, respectively (Figure 2A), the hamstring muscle force would be about 10% less. Thirdly, this model assumed that knee joint flexion moment was produced by hamstring only. Li et al. (2002) observed that the maximum knee joint flexion moment produced by gastrocnemius in the knee joint angle range from 30° to 90°was less than 3.5 Nm, which was less than 5% of the maximum knee joint flexion moment measured in the current study. Lastly, this model ignored antagonist torque produced by quadriceps. However, a previous study showed that the peak value of Rectus femoris’ activity is about 3% of MVC (Delahunt et al., 2016). Therefore, the knee joint extension moment produced by quadriceps could be ignored. Overall, this model may overestimate the hamstring force, but the magnitude would be less than 15%, even for the extreme conditions. Another limitation of this study was the relatively small sample size with large inter-individual difference. However, it seems a sizeable inter-individual difference in kinematic data is common for college level athletes (Ditroilo et al., 2013).

Our results showed F_c_ was 314.0 ± 67.8N, which is about 31–48N (344.7 ± 61.1 N for the left and 361.2 ± 65.1 N for the right side) less than that assessed by Opar et al. (2013). The difference may be caused by the relatively less body mass of participants in the current study. Buchheit et al. (2016) has observed that the contact force at the ankle hook, as assessed with the Nordbord, is large body mass (BM)-dependent (Buchheit et al., 2016). When we use their predictive equation: pFc (N) = 4 × BM (kg) + 26.1, the predictive F_c_ is no different from what we have measured. Although the study participants were not football players as recruited in other studies, the results are comparable to other studies after normalized by the body weight. Therefore, we believe the conclusion from the current study could be generalized to other populations.

In summary, the peak contact force measured at the ankle hook can only explain a little more than half (58%) of the variations in peak hamstring muscle forces during NHE. Caution must be exercised when assessing the eccentric hamstring strength using the ankle contact force during NHE.

## Data Availability Statement

The original contributions presented in the study are included in the article/supplementary material, further inquiries can be directed to the corresponding author/s.

## Ethics Statement

The studies involving human participants were reviewed and approved by the Ethics Committee of Shanghai University of Sport. The patients/participants provided their written informed consent to participate in this study.

## Author Contributions

MR initiated the idea, reviewed the literature, and drafted the manuscript. LL drafted and revised the manuscript. WZ and TH conduced the experiment. XW performed the study design and drafted the manuscript. All authors contributed to the article and approved the submitted version.

## Conflict of Interest

The authors declare that the research was conducted in the absence of any commercial or financial relationships that could be construed as a potential conflict of interest.
